# Ultrafine‐Grained Materials With Antibacterial Properties: A Novel Approach to Reducing Spinal Implant‐Associated Infections

**DOI:** 10.1002/jsp2.70091

**Published:** 2025-06-30

**Authors:** Mitsuhiro Nishizawa, Diane Hu, Hassan Serhan, Bahram Saleh, Ralph S. Marcucio, Kazuhito Morioka

**Affiliations:** ^1^ Department of Orthopaedic Surgery Orthopaedic Trauma Institute (OTI), University of California, San Francisco (UCSF) San Francisco California USA; ^2^ Rosies Base. LLC Natick Massachusetts USA; ^3^ Department of Neurological Surgery Weill Institute for Neurosciences, Brain and Spinal Injury Center (BASIC), University of California, San Francisco (UCSF) San Francisco California USA

**Keywords:** antibacterial implant, biofilm formation, implant‐associated infection, spinal implant infection, ultrafine‐grained material

## Abstract

**Background:**

Implant‐associated infection remains a serious complication of instrumented spinal surgery. Since biofilm formation on the implant surface is a key factor in the pathogenesis of such infections, current preventive strategies include the use of implants with antibiotic coatings. However, these approaches raise concerns related to antibiotic resistance and cytotoxicity. Ultrafine‐grained (UFG) stainless steel, characterized by nanoscale grain sizes, has demonstrated superior mechanical properties and potential antimicrobial effects. This study aimed to evaluate the antibacterial properties of UFG stainless steel implants against *Staphylococcus aureus* biofilm formation in both in vitro and in vivo models.

**Methods:**

UFG and conventional SUS316L stainless steel wires were incubated with bioluminescent *Staphylococcus aureus* Xen36 for up to 7 days in vitro. Biofilm formation was assessed using crystal violet (CV) staining, colony‐forming unit (CFU) counting, and quantitative PCR (qPCR) for *16S rRNA* and *luxA* genes. In vivo antibacterial effects were evaluated using two mouse models: a subcutaneous pouch model and a postoperative spinal implant infection model. Wires were harvested at 1, 3, and 7 days post‐infection and analyzed using the same assays.

**Results:**

In vitro, UFG wires had significantly lower CFU counts than standard wires at 4 h (*p* = 0.0005), 1 day (*p* = 0.0001), and 3 days (*p* = 0.0314). In the subcutaneous pouch model, UFG wires showed significantly reduced bacterial load at Day 1 by CFU (*p* = 0.011). In the spinal implant model, CFU counts were significantly lower on UFG wires at Day 3 (*p* = 0.015).

**Conclusions:**

UFG stainless steel implants demonstrated a significant reduction in early biofilm formation by *Staphylococcus aureus* in both in vitro and in vivo, suggesting a delay in the biofilm formation process. These findings support the potential of UFG materials as promising candidates for infection‐resistant spinal implants.

## Graphical abstract

This is the first in vivo study to demonstrate that ultrafine‐grained stainless steel possesses potential antibacterial properties capable of resisting the adhesion of bacteria and, subsequently, microcolony formation and biofilm maturation. Incorporating ultrafine‐grained materials in the manufacture of orthopedic implants or medical devices could uniquely balance antibacterial properties with biocompatibility, making them highly suitable for these applications.

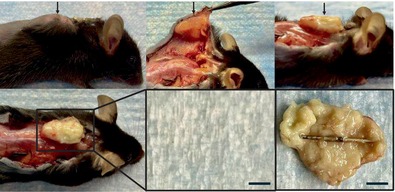

## 1 Introduction

Implant‐associated infections are a serious complication following spinal fusion surgery, leading to increased patient morbidity, mortality, and healthcare costs [1, 2]. Despite advances in surgical techniques including increased sterility levels, prophylactic antibiotic therapy, and intensive perioperative care, the incidence of implant‐associated infection following adult spinal instrumentation varies from 0.7% to 20% [3]. With the aging population driving increased demand for spinal surgeries, these infections are expected to pose a growing burden on healthcare systems [4, 5].

A key factor in implant‐associated infections is bacterial adhesion and biofilm formation on implant surfaces [6, 7, 8, 9]. Biofilms are microbial communities embedded in a self‐produced extracellular matrix, allowing bacteria to evade the immune system [10, 11, 12]. The biofilm life cycle consists of four stages: adhesion, microcolony formation, maturation, and dispersion [12]. Since preventing initial bacterial adhesion is critical to reducing infection risk, implant surface modifications have been explored as a preventive strategy [13, 14, 15, 16].

Ultrafine‐grained (UFG) materials, characterized by nanoscale grain sizes (100–1000 nm), have been developed for medical applications due to their superior mechanical strength, fatigue resistance, and biocompatibility [17, 18]. These materials can enhance cell adhesion and proliferation while modulating immune responses [18, 19, 20, 21, 22, 23, 24, 25]. However, their effect on bacterial adhesion remains unclear, with conflicting reports on whether UFG materials promote or inhibit bacterial colonization. Some studies suggest that UFG stainless steel reduces bacterial adhesion compared with conventional coarse‐grained stainless steel [23, 26, 27]. However, its antibacterial properties in vivo have not been evaluated well.

This study assessed the impact of UFG stainless steel implants on *Staphylococcus aureus* biofilm formation in both in vitro and in vivo models of implant‐associated infection. We hypothesized that UFG stainless steel would significantly reduce biofilm formation. These findings contribute to understanding the antibacterial potential of UFG materials and their implications for infection‐resistant implants.

## 2 Methods

### 2.1 Bacteria Preparation

For this work, we used bioluminescent *Staphylococcus aureus* Xen36, acquired from the American Type Culture Collection (ATCC49525) (PerkinElmer Inc., Waltham, MA). This strain harbors a genetically modified *luxABCDE* operon and a gene conferring resistance to kanamycin. These features enable us to precisely identify these bacteria using quantitative polymerase chain reaction (qPCR) to target the genes in the lux operon and to cultivate uncontaminated cultures of these bacteria in the presence of kanamycin. Xen36 was streaked on tryptic soy agar (TSA) plates containing tryptic soy broth (TSB) and 1.5% mannitol salt agar. The plates were then incubated at 37°C overnight. A single colony was selected and cultured overnight in a TSB medium with 200 μg/mL kanamycin to eliminate any contaminations at 37°C in a shaking incubator (200 rpm). The bacterial solutions were resuspended in TSB at a concentration of 1.0 × 10^8^ colony‐forming unit (CFU)/mL, determined by measuring the absorbance at 630 nm using a microplate reader (800TS, Agilent Technologies Inc., Santa Clara, CA).

### 2.2 Implant

UFG stainless steel (SUS316L) wires (Komatsuseiki Kosakusho Co. Ltd., Japan), measuring 8 mm in length and 0.5 mm in diameter, were used for both in vitro and in vivo experiments. Unprocessed SUS316L wires were used as the standard wire to compare the impacts of the UFG wires. This particular austenitic stainless steel is frequently used in various industries, including biomedical applications. A severe plastic deformation process was used to produce UFG stainless steel with a grain size of 500 nm from commercially available SUS316L, which initially had a grain size of over 10 μm. The grain size was determined using the Electron Back Scattered Diffraction Pattern detector (EBSD DigiView, EDAX LLC, Pleasanton, CA). All wires and supplies underwent autoclaving prior to the studies.

### 2.3 In Vitro Biofilm Formation

The wires were placed into the wells of a 24‐well plate containing 1 mL of a 1.0 × 10^8^ CFU/mL solution for Xen36 culture. The plates were incubated at 37°C with shaking at 200 rpm. The wires were rinsed with deionized (DI) water three times, and TSB containing 200 μg/mL kanamycin was replaced every 24 h. The wires were extracted from the medium at 4 h, 1, 3, and 7 days of incubation. At each time point, the wires were gently rinsed with DI water to eliminate any germs that were loosely attached to the surface. The biofilm on the wires was evaluated by the crystal violet (CV) analysis, the qPCR analysis, and the CFU assay as described below. All experiments were performed in triplicate.

### 2.4 Animals

All animal procedures were approved by the Institutional Animal Care and Use Committee (IACUC) at the University of California, San Francisco (UCSF). The use of bacteria was performed in a biosafety level 2 (BSL2) facility after consultation with and approval by the UCSF Biosafety Hazard Program, administered by UCSF Environmental Health and Safety. We have used BSL2 pathogens in the animal facility. Adult C57/B6 mice were used in in vivo experiments. To minimize pain, mice were anesthetized during all surgical procedures. The mice were anesthetized using inhalation of 2% isoflurane. The mice were carefully monitored after surgery for signs of discomfort, pain, or distress and administered analgesics (buprenorphine; 0.1 mg/kg) as appropriate throughout the course of the experiments or euthanized per the recommendations of the UCSF veterinary consultants. To minimize distress, mice were housed communally and provided environmental enrichment devices as allowed by Federal Regulations and our IACUC protocols. Routine care for animals was provided by the UCSF Laboratory Animal Resource Center. The euthanasia was conducted in accordance with the American Veterinary Medical Association (AVMA) Guidelines for the Euthanasia of Animals.

### 2.5 Mouse Model of Subcutaneous Pouch Infection

Seven days prior to the induction of bacterial infection, the following procedure was implemented to generate the air pouch in mice: Mice were anesthetized using inhalation of 2% isoflurane. The dorsal fur from the sacrum to the upper thoracic spine was removed with clippers. Appropriate depth of anesthesia was confirmed by ensuring that respiration remained rhythmic, slower than when awake, and unresponsive to noxious stimuli. The base of the mouse's neck was then gently pinched and lifted upward to create space between the subcutaneous tissue and the fascia. A 27‐gauge needle was used to inject 3 mL of sterile air subcutaneously into this space, thereby forming the air pouch. To maintain the cavity and mature the pouch, 3 mL of air was reinjected every 2 days. Before each inflation, the air was aspirated from the pouch to confirm the proper placement of the needle tip. Seven days after the initial air injection, the mouse was anesthetized, and appropriate depth of anesthesia was confirmed as previously described. The skin was sterilized with isopropyl alcohol and betadine. Buprenorphine (0.1 mg/kg) was injected subcutaneously just prior to the surgery, and a 5 mm midline longitudinal incision was made at the proximal end of the pouch. An implant, which included two types of wires connected vertically using a 20 μL tip cut to a length of approximately 3 mm, was gently inserted into the pouch. 3 mL of 1.0 × 10^5^ CFU/mL Xen36 culture was injected into the pouch, and the skin was closed with a wound clip and sealed with topical skin adhesive. The mice were placed on a heating pad and monitored for return to normal activity. At 1‐, 3‐, and 7‐days post‐infection, the mice were euthanized with carbon dioxide inhalation, the samples were harvested, and the biofilm on the implants was evaluated by CV assay, CFU counting, and qPCR as described below. By integrating two types of wires and inserting them as a single unit into the infection site, the subcutaneous pouch infection model allows for the evaluation of biofilms formed on each surface under the same infection conditions within the same animal [28].

### 2.6 Mouse Model of Postoperative Spinal Implant Infection

Mice were anesthetized (inhalation 2% isoflurane) and the hair on the dorsal area from the sacrum to the upper thoracic spine region was shaved with clippers. The skin was sterilized with isopropyl alcohol and betadine solution. After confirming the appropriate depth of anesthesia, a longitudinal 3 cm incision was made at the dorsal aspect of the centered lumbar spine region. By incising the fascia along the midline and carefully detaching the soft tissues and paraspinal muscles, the spinous processes on both sides were exposed. Using a 25G needle, two holes were created in the spinous process. Subsequently, wires bent into an L‐shape measuring 3 × 5 mm were employed, with the shorter arm of the pin being passed through each of these holes from the left and right sides, respectively, to anchor the implants to the spine, and the long arm extended toward the head of the mouse [29]. An inoculation of 1 × 10^3^ CFU (10 μL of 1.0 × 10^5^ CFU/mL) of Xen36 was applied in the cavity to ensure all solution contacted the implants. The fascia layer was sutured watertight with 4‐0 Vicryl, and the skin was closed using clips. Buprenorphine (0.1 mg/kg) was injected subcutaneously, and the mice were placed on a heating pad and monitored for return to normal activity. At 1‐, 3‐, and 7‐days post‐infection, the mice were euthanized with carbon dioxide inhalation; the samples were harvested. The biofilm on the implants was evaluated by CV assay, CFU counting, and qPCR as described below.

### 2.7 CV Assay

The biofilm on the implant surface was stained with CV staining and visualized with a compound light microscope [30]. Wires were gently rinsed with DI water to remove any loosely adherent bacteria from the surface. The biofilms on the wires were fixed using 100% ethanol for 1 min and then left to air dry for 3 min. Subsequently, the fixed biofilms were stained with a 0.1% CV reagent for 15 min. The stained specimens were washed with DI water and imaged using a light microscope. Then, the wires were placed in wells of 96‐well plates containing 250 μL of 33% acetic acid to solubilize the CV staining. Two hundred microliters of the suspension was then transferred to new wells, and the absorbance was measured at 630 nm using a microplate reader. All measurements were performed in triplicate.

### 2.8 Determination of Viable Bacteria Cell Number by CFU Counting

To quantify the number of living bacteria in the biofilm on the implants, we determined CFU [30]. Wires were gently rinsed with DI water to remove any loosely adherent bacteria from the surface and transferred to a 1.5 mL tube containing 200 μL of 10× trypsin [31]. After incubation at 37°C for 1 h, the wires were vortexed for 1 min and then sonicated for 10 min in a water bath with an output of 100 W. This was then followed by an additional vortexing step for 30 s to detach the biofilms from the wire surfaces and disperse them into the suspension. Each supernatant was serially diluted 10‐fold, and aliquots were plated onto TSA plates in triplicate. The colony counts were determined after an overnight incubation at 37°C.

### 2.9 Quantitative Polymerase Chain Reaction

Each wire was serially dipped three times in wells containing 1 mL of PBS to remove planktonic microorganisms and transferred to a 1.5 mL tube containing 600 μL of TSB media. The plasmid DNA was extracted from the bacteria using the Zyppy Plasmid Miniprep Kit (ZYMO RESEARCH). The final sample of DNA was eluted in 30 μL of the elution buffer, and the concentration of collected plasmid DNA was measured using NanoDrop (Thermo Fisher Scientific). Quantification was performed using a Light Cycler instrument, and the threshold cycle (Ct) was determined. qPCR analysis for the 16S rRNA and *luxA* genes was used to quantify bacterial load on the surface of the wires [32, 33]. The intensity of the luminescence signal of Xen 36 has demonstrated a reliable correlation with the total number of bacteria within biofilms in recent studies [33, 34, 35, 36], indicating that measuring the overall amount of biofilm, which includes both live and dead bacteria, is feasible by quantifying levels of the *lux* gene. The 16S rRNA gene, which is a component of the prokaryotic ribosomal 30S subunit found in all bacterial genomes, is widely used to quantify the overall amount of biofilm [37]. PCR amplification was performed in a total reaction mixture volume of 10 μL. The reaction mixture contained 5 μL SYBR Green, 1.0 μL of forward and reverse primer, 3 μL of 1:10 diluted plasmid DNA obtained from the sample, and 1 μL of nuclease‐free water. The primers used were as follows: for 16S rRNA gene, 5′‐GTGGAGGGTCATTGGAAACT‐3′ and 5′‐CACTGGTGTTCCTCCATATCTC‐3′, for *luxA*, 5′‐GAGCATCATTTCACGGAGTTTG‐3′ and 5′‐ATAGCGGCAGTTCCTACATTC‐3′. The thermal cycling protocol was as follows: initial denaturation for 2 min at 94°C, followed by 40 cycles of 15 s at 94°C, 30 s at 60°C, and 30 s at 72°C. The fluorescence signal was measured at the end of each extension step at 72°C. We generated a calibration curve, created with plasmid DNA purified directly from an overnight pure Xen36 culture [34]. The mean of the Ct values was plotted against a calibration curve to estimate the bacterial load attached to the implant.

### 2.10 Statistical Analysis

The statistical analysis was performed using IBM SPSS Statistics for Windows, version 29.0 (IBM Corp., Armonk, NY). All data are presented as mean ± standard error. The differences between the two types of wires were assessed using the two‐sided Mann–Whitney U test in all experiments. Statistically significant values were defined as *p* < 0.05. All bar and line graphs were generated using GraphPad Prism version 10.2.2 software for Mac (GraphPad Software LLC, San Diego, CA). The statistical significance of the observed differences between the two types of wires was indicated with a bar and an asterisk according to the following probabilities: *p < 0.05, **p < 0.01, ***p < 0.001.

## 3 Results

### 3.1 In Vitro Antibacterial Effect of Ultrafine‐Grained Stainless Steel Implant

**FIGURE 1:**
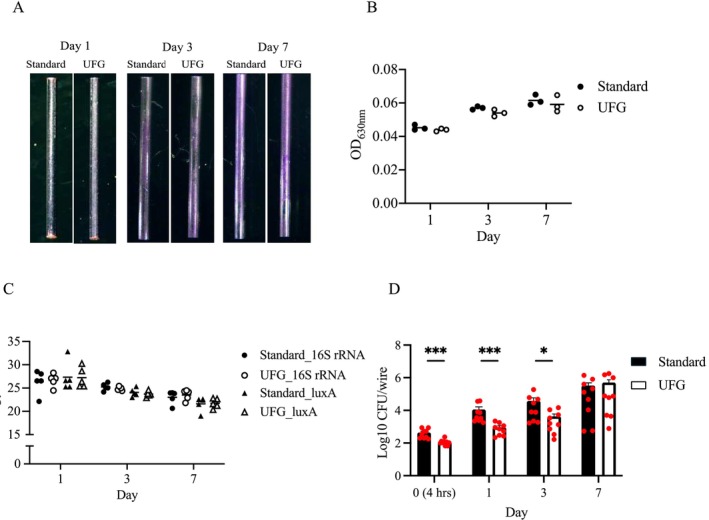
In vitro effects of ultrafine‐grained stainless steel implants on *Staphylococcus aureus* biofilms. (A) The representative images of crystal violet‐stained biofilms formed by *Staphylococcus aureus* on the surface of the ultrafine‐grained (UFG) stainless steel wire and the standard wire at 1 day (left), 3 days (middle), and 7 days (right) of incubation. (B) The absorbance measurement of crystal violet‐stained biofilms on the wire surfaces at the indicated time points (*n* = 3 per wire type). (C) The presence of the lux operon (*luxA*) and 16S rRNA genes were used to measure the overall amount of bacteria on the wire surfaces at the indicated time points (*n* = 5 per wire type). The cycle threshold (Ct) values are inversely correlated with the quantity of target nucleic acid and bacterial load. (D) The colony‐forming unit (CFU) assay was employed to quantify the total number of live bacteria present within the biofilms on the wire surfaces at the indicated time points (*n* = 9 per wire type). The horizontal line on the dot plot represents the mean values. The dots indicate individual data points. The bar graph represents the mean values ± standard error of the mean. Statistical significance was tested by two‐sided Mann–Whitney U test. *p < 0.05, **p < 0.01, ***p < 0.001.

We evaluated the biofilm that formed on the wire surfaces at 4 h, 1, 3, or 7 days of incubation. The CV‐stained biofilm became visible after 3 days of incubation and subsequently increased over time on both types of wires (Figure 1A). The absorbance of CV‐stained biofilms showed no significant differences between the two types of wires at any time points (Figure 1B). To quantify the overall amount of biofilm on the wire surfaces, we used qPCR analysis to determine the levels of *luxA* and 16S rRNA genes. Our results showed that there was no significant difference in either gene between both types of wires (Figure 1C). To determine the total number of live bacteria within the biofilm on the wire surfaces, we performed a CFU assay. We observed that the UFG stainless steel wire had significantly fewer live bacteria than the standard wire at 4 h (*p* = 0.0005), 1 day (*p* = 0.0001), and 3 days (*p* = 0.0314) after incubation (Figure 1D).

### 3.2 In Vivo Antibacterial Effect of Ultrafine‐Grained Stainless Steel Implant in Subcutaneous Pouch Infection Model

**FIGURE 2:**
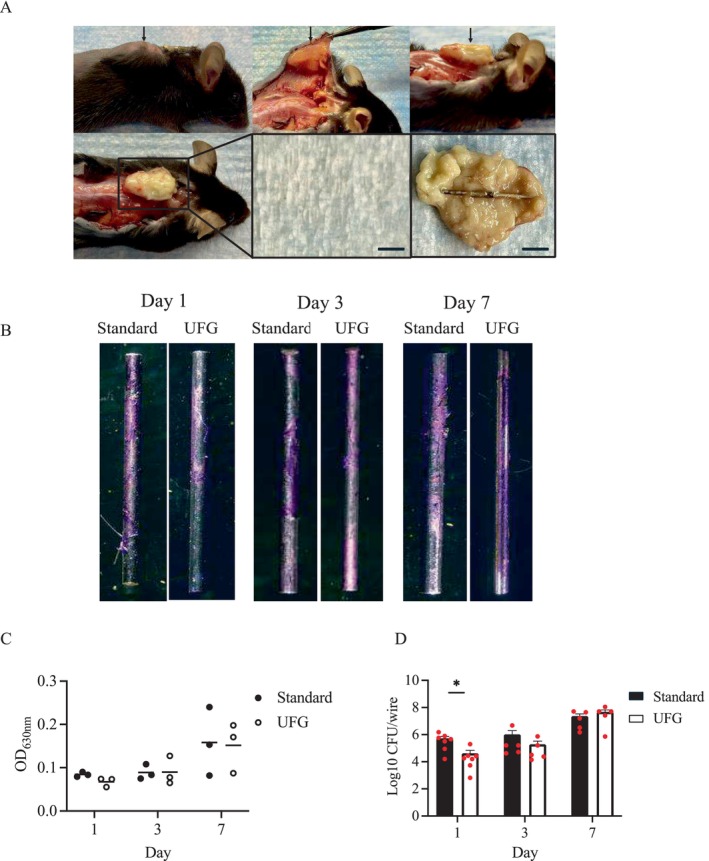
In vivo effects of ultrafine‐grained stainless steel implants on *Staphylococcus aureus* biofilms in subcutaneous pouch infection. (A) The representative image of the subcutaneous pouch infection model at 7 days after inoculation with *Staphylococcus aureus* (upper left). The well‐developed abscess was located beneath the skin (upper middle) and above the paraspinal fascia (upper right). The wire combining the ultrafine‐grained (UFG) stainless steel wire and the standard wire was present within the solitary subcutaneous abscess (lower images). The arrow indicates a subcutaneous abscess. Scale bars, 5 mm. (B) The representative images of crystal violet‐stained biofilms on the wire surfaces at 1 day (left), 3 days (middle), and 7 days (right) after inoculation. (C) The absorbance of crystal violet‐stained biofilms was measured to assess the overall amount of biofilms on the wire surfaces at the indicated time points (*n* = 3 per wire type). (D) The colony‐forming unit (CFU) assay was used to measure the total number of live bacteria within the biofilms on the wire surfaces at 1 day (*n* = 7 per wire type), 3 days (*n* = 5 per wire type), and 7 days (*n* = 5 per wire type) after inoculation. The dots represent individual data points, and the mean values ± standard error of the mean are shown in the bar graph. Statistical significance was tested by two‐sided Mann–Whitney U test. *p < 0.05, **p < 0.01.

Following incubation, a fully developed abscess encasing both wires formed in the subcutaneous region (Figure 2A). The CV‐stained biofilms were detected 1 day after inoculation (Figure 2B) and were not different between the wires at any time point (Figure 2C). However, the UFG stainless steel wire had significantly fewer live bacteria than the standard wire 1 day after inoculation (*p* = 0.011; Figure 2D).

### 3.3 In Vivo Antibacterial Effect of Ultrafine‐Grained Material in Spinal Implant Infection Model

**FIGURE 3:**
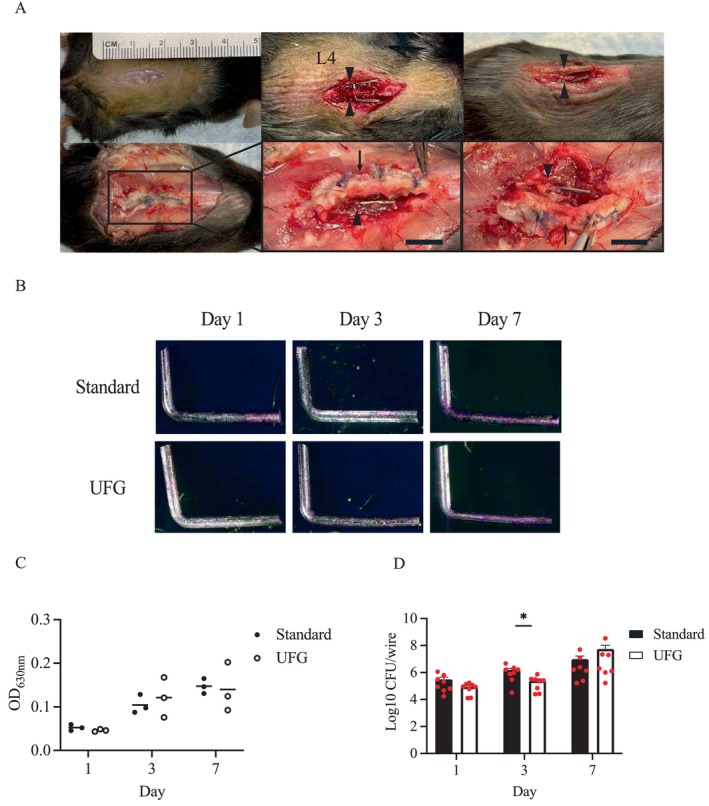
In vivo effects of ultrafine‐grained stainless steel implants on *Staphylococcus aureus* biofilms in spinal implant infection. (A) The representative images of the spinal implant infection model. To expose the spinous process of the fourth lumbar vertebra (L4), a skin incision was made in the middle of the lumbar spine (upper left). After two holes were created in the spinous process, an L‐shaped ultrafine‐grained (UFG) stainless steel wire or an L‐shaped standard wire was inserted into the hole from either the left or right side (upper middle, right). The extensively formed abscess was located at the paraspinal fascia (lower left) and above the vertebrae (lower middle, right) at 7 days after inoculation with *Staphylococcus aureus* . Both lateral wires were present adjacent to the abscess (lower middle, right). The arrow denotes a subfascial abscess, and the arrowhead indicates a wire. Scale bars, 5 mm. (B) The representative images of crystal violet‐stained biofilms on the surfaces of the L‐shaped wire at 1 day (left), 3 days (middle), and 7 days (right) after inoculation. (C) By measuring the absorbance of crystal violet‐stained biofilms, the overall amount of biofilms on the wire surfaces was evaluated at the indicated time points (*n* = 3 per wire type). (D) The total number of live bacteria within the biofilms on the wire surfaces was quantified by the colony‐forming unit (CFU) assay at 1 day (*n* = 8 per wire type), 3 days (*n* = 8 per wire type), and 7 days (*n* = 7 per wire type) after inoculation. The dots in the graph indicate individual data points, whereas the bar graph displays the mean values ± standard error of the mean. Statistical significance was tested by two‐sided Mann–Whitney U test. *p < 0.05.

After inoculation with Xen36 into the right and left subfascial spaces, an extensively formed subfascial abscess developed in the midline of the lumbar spine over time, but both wires were not completely encased in the abscess (Figure 3A). The presence of biofilms was observed beginning on 1 day after inoculation (Figure 3B) and were not different between wires at any time point (Figure 3C). However, again, the UFG stainless steel wire had significantly fewer live bacteria than the standard wire at 3 days (*p* = 0.015) (Figure 3D).

## 4 Discussion

In this study, we found that UFG stainless steel materials have the potential to resist the early stages of biofilm formation when exposed to high amounts of *Staphylococcus aureus*, both in vitro and in vivo, even in the absence of antibiotics. Considering that biofilm formation occurs rapidly [35], delays in the biofilm formation on the UFG stainless steel wires may confer an advantage by slowing this process.

For in vivo assessments, we evaluated the effects of UFG stainless steel implants on biofilm formation using a subcutaneous pouch infection model and a modified lumbar spinal infection model [29, 36]. In general, as in vitro biofilm formation is highly dependent on the conditioned medium in the absence of the immune system [37], the direct comparison between in vitro and in vivo results is challenging [38]. Our experimental models were created to mimic the clinical characteristics of implant‐associated infection following instrumented spinal surgery such as the development of an abscess around implants, and allow for the evaluation of biofilm on two types of implants within the same individual and under identical infection conditions.

Although the trend was consistent with that observed in vitro, the two in vivo models showed differences in the timing of significant CFU reductions. In the pouch model, fewer live bacteria were detected on Day 1, whereas in the spinal model, a reduction in bacterial counts on the UFG wires was observed on Day 3. The differences in these results could be attributed to several factors, including the location of the infection site, the volume of bacterial inoculation, and variations in the biofilm formation process. In vivo, *Staphylococcus aureus* biofilm development typically progresses through distinct phases: initial attachment by planktonic bacteria on Day 0–1, robust bacterial proliferation on Day 3, and maturation with increased biofilm matrix formation by Day 7 [39]. However, it is reported that the initial adhesion phase of biofilm formation is influenced by bacterial load. In the pouch model, the wire is completely surrounded by abscess tissue, and the bacterial load is relatively high. In contrast, in the spinal model, the abscess primarily extends dorsally to the wire, and the volume of bacterial inoculum is much smaller. Given that UFG appeared to exert its effects during the early stages of biofilm formation, potential explanations included a delayed progression from attachment to proliferation in the spinal infection model, where the bacterial burden is lower compared to the pouch model.

The delay in establishment of the biofilm UFG stainless steel may have significant clinical value. The lower levels of contaminating bacteria in a clinical setting may have less chance of establishing a biofilm on the implant in the presence of prophylactic antibiotics and the immune response. Moreover, when combined with current antibiotic strategies, UFG stainless steel may enhance the antibacterial properties [40, 41]. In addition to their antimicrobial potential, the superior strength, fatigue resistance, and enhanced biocompatibility of UFG stainless steel suggest that these materials could serve as multifunctional implants, reducing not only the risk of infection but also mechanical complications, pseudarthrosis, and allergic reactions [17, 18, 42, 43, 44].

This study has several limitations. First, the investigation was conducted using only one strain of *Staphylococcus aureus* , Xen36, and a single type of UFG material, stainless steel UFG with a grain size of 500 nm, which is produced by the warm oval square rolling process. The bacterial cell behavior and the subsequent inflammatory response can vary depending on the grain size, nanoscale topographic roughness, and the size and shape of the implant surface, the bacterial species, and even the specific process to create the UFG materials [23, 24, 25, 26, 27]. Secondly, this study only compared biofilm formation at relatively early time points, up to 7 days post‐infection. Therefore, the effects of UFG materials on bacteria at later time points remain unclear, and evaluating these later stages is crucial for considering the application of UFG materials in orthopedic implants. Fourth, we used very large bacterial inoculums which may not reflect a clinical scenario, and we did not provide prophylactic antibiotics or treat the infections as would be standard of care. Fifth, although we carefully rinsed the wires before conducting CV staining, CFU, and PCR assays, these methods cannot strictly differentiate between bacterial biomass or bacterial cell clusters and biofilm. Therefore, the possibility remains that some measurements may reflect them rather than biofilm. Lastly, this study did not evaluate the mechanical or biomechanical properties of the metal, including surface roughness, surface charge, and wettability, in conjunction with the biofilm assessment. Comprehensive mechanical, biomechanical, and biomaterial evaluations are required for the future application of UFG materials in spinal implants.

In conclusion, to our knowledge, this is the first in vivo study to demonstrate that UFG stainless steel possesses potential antibacterial properties capable of resisting biofilm formation. Incorporating UFG materials in the manufacture of orthopedic implants or medical devices could uniquely balance antibacterial properties with biocompatibility, making them highly suitable for these applications.

## Author Contributions

H.S., B.S., R.S.M., and K.M. contributed to the conception and design of the study. M.N. and D.H. performed material preparation, experiments, data collection. M.N. and K.M. performed data analysis. M.N. wrote the original draft of the manuscript. R.S.M. and K.M. revised the manuscript. All authors reviewed and approved the final version of the manuscript.

## Conflicts of Interest

The authors declare no conflicts of interest.

## Data Availability

The datasets generated and analyzed during the current study are available from the corresponding authors upon reasonable request.
